# How do users with comorbidity perceive participation in social services? A qualitative interview study

**DOI:** 10.1080/17482631.2021.1901468

**Published:** 2021-03-22

**Authors:** Amanda Jones, Kari Jess, Ulla-Karin Schön

**Affiliations:** a School of Education, Health and Social Studies, Dalarna University, Falun, Sweden; b Department of Social Work, Stockholm University, Stockholm, Sweden

**Keywords:** Participation, user knowledge, substance use, mental illness, grounded theory

## Abstract

**Purpose**: This study aims to construct a theoretical framework that explains how users with comorbidity of substance use and mental illness/neuropsychiatric disorders portray user participation in social work encounters.

**Methods**: To construct this framework a constructivist grounded theory approach was used with semi-structured qualitative interviews with 12 users.

**Results**: The main concern of the participants was the low trust in the social services and perceiving that this lack of trust is mutual. Establishing mutual trust is a social process that cuts through the whole framework. In the framework, prerequisites for participation are explained. The prerequisites are users being motivated and having the willingness to stop using drugs and receiving support, making use of user and staff knowledge and decision-making abilities and accessing help and support.

**Conclusion**: Unlike previous frameworks, the model describes participation as a social process and does not explain participation at different levels of power. The results suggest that staff need to be aware of low trust perceptions and work on establishing mutual trust. In addition, the staff need to see each user as an individual and consider how the user would prefer to be involved in decision-making.

## Introduction

People receiving support from the social services (i.e., users) have the right to participate in the treatment planning and decision-making (Beresford & Croft, 2004; Vedung & Dahlberg, 2013). User participation has its roots in the principles of democracy and citizenship. Leaning on these principles, user participation during the past decades has been emphasized in legislations, social care policies and codes of ethics in many Western countries (Askheim et al., 2017; Beresford & Branfield, 2006; Björk, 2016; Blennberger, 2017; Carr, 2007; Eldh & Winblad, 2018; Eriksson, 2015; Nykänen, 2020; Rutter et al., 2004). In addition, user preferences and wishes are highlighted in an evidence-based care practice (EBP) additional to staff (i.e., staff in the social services) knowledge and skills, research evidence and other relevant circumstances (Bergmark et al., 2011; Haynes et al., 2002). Reasons for emphasizing user participation in legislation and policy documents are derived from shifting values and attitudes, as well as advances in research showing the positive consequences of user participation (Eriksson, 2015).

Comorbidity refers to the state of having two or more diagnoses at once. In this article comorbidity refers to co-occurring substance use and mental illness/neuropsychiatric disorder. Within mental healthcare and substance misuse treatment, user participation can increase treatment satisfaction (Brener et al., 2009; Storbjörk et al., 2016) and lower dropout rates or longer retention rates (Graff et al., 2009; Laugharne & Priebe, 2006; Swift & Callahan, 2009). Nevertheless, when it comes to efficiency and outcome measures, the results are inconclusive (Storbjörk et al., 2016) but the general view is that user influence has a positive effect on the treatment outcome (Swift & Callahan, 2009). For example, there is evidence that user influence in treatment planning positively affects the achievement of treatment goals (Brener et al., 2009). Participating in decision-making processes through shared decision making has also resulted in a decrease of psychiatric symptoms, less drug use and an increase of autonomy among users with comorbidity of mental illness and substance misuse (Joosten et al., 2009, 2011).

Notwithstanding these positive effects, the implementation of user participation in health and social services is limited (Eriksson, 2015; Karlsson & Börjeson, 2011; Nykänen, 2020). Some of the acknowledged difficulties in implementing user participation are a discrepancy between policy and practice (Eriksson, 2015; Laitila et al., 2011) and staff attitudes (Laitila et al., 2011; Schön et al., 2018). In policies the designations and definitions of user participation lack consensus on how user participation should be implemented in practice and what it entails (Leung et al., 2020; Levin, 2012; Nykänen, 2020). The understanding of user participation may vary depending on if users, politicians, chiefs, or staff are asked (Eriksson, 2015; Rutter et al., 2004; Thompson, 2007). The differences primarily reflect the degree of real influence and power for the user as participation can involve anything from participating in a process to having an equal partnership between users and staff (Nykänen, 2020; Sahlsten et al., 2008; Vedung & Dahlberg, 2013).

In some practices user participation is complicated and goals such as partnership and self-determination are jeopardized by low autonomy due to misuse or the need for coercive action. Studies have shown that both users with addiction problems and users with mental health problems often lack the opportunity to participate in decision making and planning of treatments (Bee et al., 2015; Harris & McElrath, 2012; SOU, 2011, p. 35). One reason for this is that their ability to participate and have an impact is questioned by staff (Laitila et al., 2011). Moreover, users receiving substance misuse treatment experience a lack of autonomy and flexibility (Ekendahl & Karlsson, 2016). There is also a risk of having to confront paternalistic views and prejudicial attitudes from staff (Laitila et al., 2011). Moreover, users with substance misuse proclaim that they are encouraged to be passive recipients of treatment (Harris & McElrath, 2012).

The wide variety of needs and shifting abilities among users in social services are obstacles to implementing user participation (Knutsson & Schön, 2020). In line with this argument, substance misuse and certain types of mental illness can lead to an impairment of the individual’s decision-making ability (Kovacs et al., 2017; Mallorquí-Bagué et al., 2016; Spencer et al., 2017). In addition, different stages of addiction, such as withdrawal, cravings, intoxication and compulsive substance use can affect a person’s ability to reflect and think clearly (Spriggs, 2003). For this reason, the need to account for the users’ perspectives on their expectation of participation and how they want to be involved in encounters with social services is emphasized (Friedrichs et al., 2018).

Today, there is a lack of knowledge on how users with a perceived weakened autonomy (such as comorbidity) regard participation. By exploring this phenomenon, knowledge can be gained on how users explain participation and how this can be understood. This explanation can adjust user participation in social services to meet the users’ needs and improve treatment efficacy. Although user participation has its roots in the principles of democracy and citizenship, it is a concept put forward by lawmakers and politicians as a top-down expectation on services and providers (Beresford, 2002, 2003; Eriksson, 2015). Exploring the users’ views on what user participation entails, may enforce a bottom-up, democratic approach (Beresford, 2003; Hui & Stickley, 2007) of participation. Moreover, exploring users’ views can contribute with important knowledge to reach consensus on the implications of the concept and promoting the implementation of user participation. This exploration could strengthen the user’s voice in social work encounters and facilitate partnerships. Thus, this study seeks to explore how users perceive participation in social work encounters through a grounded theory approach.

## Methods

A constructivist grounded theory (CGT) approach (Charmaz, 2014) was used to explore users’ subjective experiences and create a theoretical framework on what user participation entails and the underlying processes of participation. There are several grounded theory orientations available (see e.g., Charmaz, 2014; Corbin & Strauss, 2015; Glaser & Strauss, 1967). One of the differences between the orientations is how data is viewed; from being objective according to Glaser (2002) to being constructivist according to (Charmaz, 2014). In the present study Charmaz CGT was used since data is not viewed as objective, rather that the model is constructed in the process of collecting and analysing data. In contrast to earlier Grounded theory rules stating that one should analyse data without preconceived ideas, Charmaz acknowledges that everyone has a pre-understanding or expectation. It is therefore important to reflect upon how a pre-understanding influenced the study and how it can be properly handled (Charmaz, 2014). The authors of the paper have a combined knowledge regarding both clinical work and conducting research within the field of study. For example, this has influenced the process of creating the initial open-ended interview guide and facilitated the recruitment process. However, to make sure the constructed model is grounded in the collected data, not in pre-conceived ideas or assumptions, the authors have had continuing discussions regarding study design and during the data analysis.

The theoretical framework explains how users with comorbidity perceive participation in social work encounters, particularly in encounters with those in a decision-making position, and is grounded in semi-structured interviews.

### Recruitment

Recruitment was done through three sources to reach users at different stages of substance abuse and experience: staff in the social services (social workers in a decision-making position and inpatient and outpatient social workers), health care providers and user organizations. Staff within these services were contacted by email and an information letter was distributed. Staff asked potential participants whether they wanted to receive more information about the study and whether they were willing to participate and then provided contact information to the first author (AJ). Subsequently, AJ contacted potential participants and provided them with verbal information about the study and the study’s purpose. Potential participants were also offered an information letter before the interview took place. Before the start of the interview, the participants were asked to read the information letter and sign a consent form.

Inclusion criteria were: 18–65 years of age, previous or ongoing contact with the social services due to comorbidity of substance misuse and mental illness/neuropsychiatric disorders. Exclusion criteria were active substance abuse and mental illness only caused by substance abuse.

### Participants

Fourteen potential participants were contacted and 12 consented to participate (9 men and 3 women, aged 22–65 years). Most respondents had ongoing contact with social services during the interview period. Overall, the respondents had extensive experience of in- and outpatient treatment and housing support. Participants had a variety of mental illnesses, including depression, anxiety, post-traumatic stress disorder (PTSD) or bipolar disorder. For neuropsychiatric disorders, participants experienced attention deficit hyperactivity disorder (ADHD), attention deficit disorder (ADD), or autism spectrum disorder. In addition, some of the participants had comorbidity of mental illness and neuropsychiatric disorder. Two participants strongly suspected that they had an ADHD/ADD diagnosis. These two participants also said that staff suspected that they had a diagnosis of ADHD/ADD. Moreover, there was a variation of substance misuse with alcohol misuse, illicit drug use, or a combination of alcohol misuse and illicit drug use. In all, there was variation in age, sex, form of housing, mental illness, neuropsychiatric disorders, substance misuse and how the respondents view the social services distributions.

### Data collection and analysis

Given that CGT is an iterative method with an interplay between data collection and analysis (Charmaz, 2014), collection and analysis of data have been done in parallel. The interviews were conducted between October 2019 and April 2020. All interviews were held by the first author (AJ). As recommended in CGT, a semi-structured interview guide with four central themes (participation, information, response, assessment) was initially developed. The interviews were recorded and transcribed verbatim, as recommended in CGT (Charmaz, 2014). All interviews, lasting between 38 and 72 minutes, were included in the analysis. Each theme consisted of two or three open-ended questions followed by a question of whether the participants had any additional items to address.

Coding and categorizing transcripts were done in the software program NVivo v. 12 plus. Interviews 1–7 were conducted using the initial interview guide, and interviews 8–12 were held with a revised interview guide based on the coding of interviews 1–4. This arrangement is in line with the notion in CGT, i.e., what is being studied is continuously evolving and takes new turns that the researcher needs to sustain (Charmaz, 2014).

Data and categories were interacted during the coding process, i.e., a movement back and forth between being close to the data versus the theoretical level. All data in interviews 1–4 were coded (Charmaz, 2014). This initial coding was mostly done nvivo (using the participants’ own words) and smaller tentative categories were constructed. The analysis of interviews 5–7 led to a re-categorization of the tentative categories in which different codes and tentative categories were placed together into new tentative categories. Interviews 1–4 were therefore recoded based on the new tentative categories. After that, the whole coding process was again reviewed for consistency. Discussions among all authors concerning codes and subcategories were held throughout the analysis process.

The coding of interviews 8–12 was conducted in conjunction with the revised coding. However, during a continued memo-writing process in which categories and sub-categories were covered to determine whether (and how) they are linked, some former sub-categories were combined. All interviews and codes were then read to determine whether anything needed to be adjusted or was missing a code. The model constructed in the analysis was during this process of memo-writing and discussions shaped. In the model, four prerequisites of user participation compose the core categories, besides the main category of establishing a mutual trust which cuts through all the other categories. At this point, we made the judgement that further data collection was unnecessary since additional information would not influence the categories.

The study was approved by the Swedish Ethical Committee (no. 2019–00657 and complement no. 2019–03971). In accordance with ethical guidelines Swedish Research Council (n.d.) all participants are anonymous and pseudonyms are used in the result section.

## Results

A conceptual framework of *“A social process of establishing a mutual trust”* was constructed through the analyses process. The main concern that was delineated from the interviews was a perception of low mutual trust between users and staff in social services. The social process of establishing mutual trust therefore cuts through the process of establishing user participation. In this process users and staff are co-creators in which the users place responsibility on themselves and the staff to improve mutual trust. The purpose of the social process is to reach shared decisions on treatment and support and to implement them jointly.

The results below are initially presented detailing the model’s significance in its entirety, followed by a more detailed description of the core categories.

**Figure 1.**
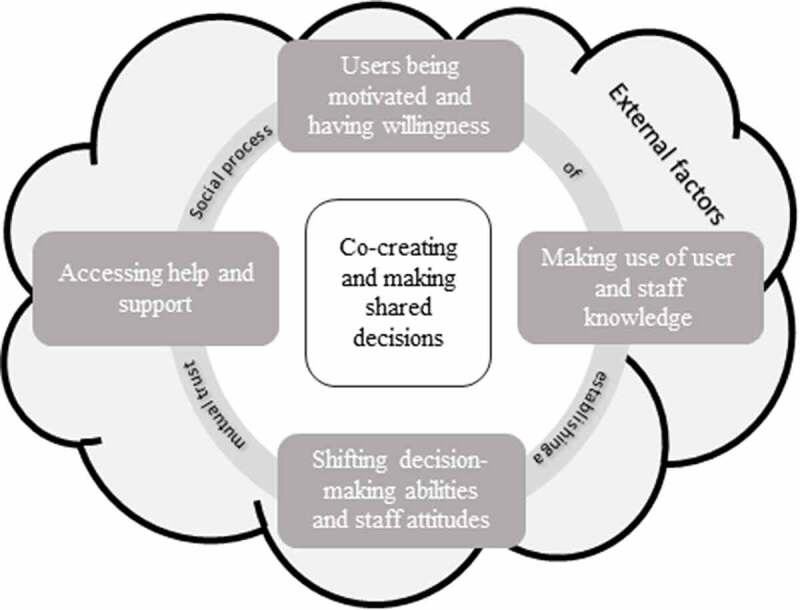
Illustration of prerequisites of user participation in social work encounters

Figure 1 illustrates the four core categories as prerequisites for establishing user participation as a social process with mutual trust. The basis of participation is that the users are being motivated and having willingness to end the use of substances and making use of user and staff knowledge. In addition, users’ decision-making ability is described to be shifting. This shifting decision-making ability has to be accounted for by staff having the ability and intent to create opportunities for users to participate in decision making. Another factor in the process of participation and establishing mutual trust is user access to help and support. These prerequisites contributed to a perceived co-creation and shared decision making in social work encounters.

In addition, decision making is influenced by external factors regarding the conditions of users and staff for user participation. For example, participants explained that the user network could not only facilitate but also hinder recovery. A supportive network that can follow the users to social service meetings is seen as facilitating. On the other hand, having people around you that are still using drugs is considered a hindrance. Respondents feel that having contact with organizations working with those with substance use or a criminal record is an external facilitator. Participants were cognizant that limited budgets could hinder decisions that both users and staff would prefer. In addition, the hierarchical system in the social services was outlined as a possible obstacle to shared decision-making, given that users at times meet with staff who do not have the formal authority to make decisions. Moreover, according to the participants, laws and routines that staff need to follow as well as staff turnover may hinder the decision-making process.

### Mutual trust

As noted above, mutual trust is a core factor that cuts through all categories. Having had the opportunity to work together with staff through decision-making leads to feelings of involvement and the perception of equality and mutual trust. Participants also note the importance of equality, i.e., an equal sense of power, with staff.

The participants explained entering encounters with social services with an initial low trust, being “lucky” when meeting good staff. The respondents’ low trust came from several sources: previous negative encounters, internal trust issues, or an overall critical image of social services. This lack of trust is perceived as reciprocal in that participants feel that staff also have a low initial trust of users.

However, establishing mutual trust is a social, reciprocal process where participants also reflected on their contribution, illustrating an awareness of their sometimes manipulative behaviour and how this may influence staff trust.

First of all, it feels like they [staff] think you are lying about everything when you are active in substance-use.

Eh, and that is something you do pretty much. But one [staff] cannot assume that a person always lies because

there come to these few occasions when you are sincere and really need the help you ask for. But it is

understandable also, when you do as you do with the disease [addiction]. But . but for example, when I tried

to explain my side of it when I was beaten [by the child’s father] and thrown out [from their mutual home]

and stuff like that. Then they [staff] did not listen to me at all. (Anna)

Participants described that trust in staff was important as it encourages users to express their views, thoughts and preferences. Trusting also improved their understanding of the actions of social services which did not correspond to their wishes, e.g., decisions pertaining to mandatory treatment. Circumstances highlighted as facilitating trust were having one destined and available member of staff, receiving support beyond the perceived formal obligations and staff not giving up on users in their times of failure or during special support needs.

Participants also described specific staff characteristics as important, including being modest, patient, emphatic and understanding, having faith in users and waiting for users to accept support while not pushing for it. Being overly assertive may lead to non-functioning support, which is seen as a waste of resources because users’ will and motivation are essential.

### Users being motivated and having willingness

Another fundamental condition for user participation is user motivation and willingness to stop substance use and accept support. The participants explained that without personal will and motivation, support was fruitless. Manipulation is described in most of the interviews and aims at enabling a continued substance use.

Eh, before, I manipulated authorities, misbehaved, all for my personal gain because [at that time] I was in

heavy substance abuse. The only thing I thought about was drugs, drugs, drugs. So my whole criminal

mindset was used on all possible fronts, even with the social [services] where I like maybe manipulated them

to give me extra money or things like that (Jacob)

In the interviews the participants emphasized the need for an overall reason to decide to terminate the use of drugs. The decision demands taking responsibility, seeking support and being more honest with oneself and staff. Several reasons for this decision are illustrated in the interviews and seem to derive from the negative consequences substance use has had on the participants’ lives.

The participants explained that by having the will and motivation to change, their behaviour in turn, changed from withdrawing from the social services to taking responsibility and seeking support. From being manipulative towards staff, they went to great lengths to be as honest and “laid all cards on the table”. Honesty was described as a presumption of receiving the right kind of support, or in some cases, could be a “scream for help”. The participants described how trustworthiness was manifested by taking responsibility, following care plans and participating in activities. Yet, it also meant taking responsibility for their situation by not buying drugs to the same extent as earlier, or by being honest with the staff, especially regarding relapses. However, motivation may be temporary and fragile, and the participants emphasized the importance of being heard and receiving support when there were self-regulation, will and motivation.

### Making use of user and staff knowledge

A second basic condition for user participation concerns user and staff knowledge. The participants possessed an in-depth knowledge of themselves, their needs, reasons for their substance use, how they act in different situations and their strengths and weaknesses. They also have a general awareness of substance misuse and mental/neuropsychiatric disorders and how they are affected by comorbidity. This subjective experience-based knowledge brings about user preference and treatment proposals. Participants explained that staff need to seek and use this experience to gain user trust. It is about staff asking for the users’ experiences of their addiction, what helps and how they experience the interventions that are offered, i.e. that they consider the user as a bearer of knowledge.

“A girl who was, she had probably just finished the education, she asked if she could interview me once a

week for several weeks one spring. “Yes, go for it”. But why did I ask her. “I have no experience with this, I don’t really work with this”. So I asked but why are you going to do this? “It is because I like to broaden [my knowledge]”. So that was a good thing.” (Karl)

In addition, user knowledge contributed to staff knowledge by users giving information about their situation. From this information users experienced that staff made an assessment regarding their situation to give the right support. The participants perceived that extensive knowledge of user needs and difficulties was vital to adjust the approach and support in accordance with the user’s situation, needs and preferences.

They [staff] need to hear me, and be able to understand. That is, if it is substance use and like me with diagnoses, I have PTSD and people [staff] who do not know what PTSD is, they cannot understand why I am like I am, nor can I get help then by someone who does not know what problems I have. (Anna)

### Shifting decision-making abilities and staff attitudes

The interviews revealed that power of users and staff to make a shared decision influences decision-making. During and for a period after active substance misuse, it may be difficult for users to recognize their needs, consider alternatives and make decisions. Also, their mental illness may affect their ability to make a decision. For staff, it is about how to involve users in decision-making processes and attitudes to users’ ability to be active in such processes.

The aspect that participants most emphasized is that the staff listen to the users to gain experience-based perspectives of their health problems and social situation. This aspect includes the feeling of being heard despite the result this has on the decision-making process, or that their voice influences decision making. The latter is more important in having the possibility to participate, whereas the former is important regarding the service user’s feelings of being respected and taken seriously. Being listened to and experiencing what is said have an impact on the decision, is also described as necessary in the recovery process.

Yes, and that was what saved me when I went through the addiction programme. I have had a lot of psychologists, contacts and similar but none of them have worked because they have followed these routines that they have, rather than looking outside the box, but then I got someone who listened to what I said, really. How to explain it … really saw me as a person and listened to what I had to say. Instead of going after her feelings, she went after mine and it was a huge help (Anton)

Being ignored can negatively affect user trust for staff, leading to the users not speaking up for themselves or expressing anger or irritation towards the social services.

At least being heard goes a long way actually. Not being heard is, even if, even if one is heard and the result of it is perhaps not what you wanted or thought. At least being heard goes a long way. Not being heard can destroy a lot at the moment, I believe. Me, as many others in that phase already thinks, “to hell with authorities”, “to hell with normal people”. They don’t know crap and so forth. That is when you need help and a push in the right direction. Not being heard at that moment will only exacerbate the anger one has, or the contempt one has towards authority, and so on. That is something quite dangerous. (Jacob)

Collecting information from others and presenting proposals to staff are examples of how users contribute to decision making. Respondents also experience information from staff as important (e.g., regarding plans that are being made and receiving this information before the actual meetings take place). Yet, this information needs to be clear and accurate. Incorrect information may affect users’ trust in the staff.

In many cases they [staff] say that it is going to be a meeting to plan but then it is a decision-making meeting. This is what they should not be allowed to do, as I see it, to say that the meeting is going to be one thing and then it is something entirely different (Anton).

Similarly, information on user rights and different treatment options is critical to facilitating a shared decision-making approach. Nevertheless, there is an awareness of temporary difficulties in being involved because of mental illness or episodes of substance misuse. When users have these temporary difficulties to participate in decision making, it becomes an imperative for staff to make the necessary decisions. When this occurs, users must trust the staff to give them the mandate to make decisions. Staff making choices during these times was not experienced as power oppression, but rather as a feeling of “security”. For instance, when staff need to make decisions about mandatory treatment to protect users from a life-threatening situation, it was described as something needed and the users understood why they were made.

Discussions about alternatives are considered potentially important, although this depends on the users’ decision-making ability. Being involved and to get the opportunity to try different treatment options before deciding which one was seen as positive. Informed discussions on treatment options were also important during treatment (e.g., regarding prolongation of the treatment). For users who were ambivalent about whether to quit using drugs, being able to discussing pros and cons with different options was deemed positive. Consequently, introducing and discussing options with users viewed as a way to help the user get ahead in the process. When the user has the motivation and the decision-making process is ongoing, the commissioning and provision of care and support need to be readily available.

### Accessing support

A presumption of access to support was staff availability in terms of easily accessible by phone or other means and staff having flexible work hours. A perceived understaffing and short designated telephone hours were by many respondents described as obstacles to user involvement.

Obstacles [for user participation] is that they [staff] are too few and they are not allowed to meet [users] too often. Rather, many times, instead it is these designated telephone hours between 09.00 to 09.45 and then it is closed and you can’t reach your social worker. And during these times, the same social worker for sure has 30–40 other [users] that need to talk. (Jonas)

Access to support also concerned staff continuity, which included long-term planning and clear transitions to new staff when regular staff quit or are on sick leave. Another aspect of access to support was that staff offered personalized support within a reasonable time frame and if more caregivers were involved to coordinate the support programme. Lack of support led users to try to convince the staff about their needs by using escalating or persuasion tactics. Users would try to persuade the staff to provide support through various potentially dangerous behaviours.

I have had problems regarding getting the chance to go to treatment facilities. The last time it took almost five months […] and they didn’t let me. So, I had to drink more and more and, at last, I tried to commit suicide and then I got to go away. (Tord)

Participants reported that periods of motivation in accepting support might be brief, making it all the more important to get support as soon as possible. Quick access to support was also viewed as grounds for users to feel that they got help. One reason mentioned for not receiving urgent support was that staff do not fully trust the users.

Where users have contact with more than one service, staff coordination is most important. This coordination can be accomplished, for instance, through coordinated meetings where users meet with all caregivers at the same time, sharing the same information and coordinating care plans. However, from the interviews, adaptations to these meetings need to be done to ensure that the users understand each staff member’s area of responsibility and have the courage to speak up (e.g., in the interviews it was mentioned that users with problems speaking in group discussions are likely to be silent and prefer smaller meetings). Other adaptations suggested concern for the support itself in terms of user needs.

However, one major obstacle to support is if the user is not sober. Some participants noted that staying sober from substance misuse determines the possibility of receiving support. The situation is described as paradoxical because respondents seek support to get sober.

## Discussion

This study sought to construct a model to explain how users with comorbidity of substance use and mental illness/neuropsychiatric disorders depict user participation in social work encounters. A limitation with respect to CGT is that theoretical sampling was not used. The reason for this was the limited access to participants, which is why all users willing to take part in the study were included. It was not possible to choose between users other than by applying the inclusion and exclusion criteria. Still, there were variations on the type of substance use and mental illness/neuropsychiatric disorders, duration and content of social services support and views of the social services, which is why we deem the sample valid. Another limitation is the relatively small sample size (n = 12). However, recruitment and analysis were done in parallel and sampling was conducted until the categories were filled and no additional information was considered necessary.

Nevertheless, the present study contributes with valuable knowledge in the efforts to implement participation in social services via a bottom-up, democratic approach (Beresford, 2002, 2003; Eriksson, 2015) by adding knowledge on users views of how participation can be understood. The model offers some insight into how users portray participation, which can be seen as a valuable contribution to a bottom-up democratic approach (Beresford, 2003; Hui & Stickley, 2007). The current theoretical framework illustrates user participation as a social process between staff and users and shows how establishing mutual trust, sharing knowledge and being available are essential parts of user participation. The model illustrates how co-creating and sharing decisions require mutual trust between staff and users. Through the process of establishing mutual trust, users may still perceive participation in decisions although their decision-making ability is sometimes weakened.

Previous frameworks of participation (see, e.g., Arnstein, 1969; Shier, 2001) focus on power and illustrate participation as a step-by-step ladder: from no participation, to tokenism to equal power. These frameworks are rooted in the democratic approach to participation (Beresford, 2003; Hui & Stickley, 2007) with an overall focus on power and social rights (Arnstein, 1969). The current model also reflects a democratic approach. Yet, instead of focusing on levels of power and power distribution, the framework depicts a social and relational process. Through this process, mutual trust is sought to facilitate participation and shared decision making in social services practice. Stories of power are expressed in the interviews but are not a parallel process in building mutual trust and participation. Rather, participants seem to express a feeling of power even in times of low participation, e.g., by manipulating staff, getting their will across and stating that their own willingness to end the use of substances is a prerequisite for the encounters.

The model also underscores the importance of making use of user knowledge. Participants expressed knowledge about themselves and their needs, as well as general knowledge of substance use and mental illness/neuropsychiatric disorders. Participates also discussed external factors hindering participation in social work encounters, such as the social services laws and routines and strict budgets, indicating that they also have an understanding of the conditions of the social services. The undeniable value of practical knowledge in social work encounters has been expressed in previous research (Knutsson & Schön, 2020; Laitila et al., 2011). From that perspective, the broad user knowledge illustrated in our model becomes an asset to social service collaboration. At the same time, the results jeopardize the idea that clients with comorbidity are unable to participate due to poor decision-making skills (Kovacs et al., 2017; Mallorquí-Bagué et al., 2016; Spencer et al., 2017). In line with this, in EBP, staff who consider *user knowledge* and *user preference* and values (see, e.g., Bergmark et al., 2011; Björk, 2016; Haynes et al., 2002) may enhance user involvement in evidence-based social practice. Otherwise, the starting point will be that the collaboration consists of only one knowledge bearer, neglecting that users have equally valuable knowledge.

The results provide valuable insights on how user participation can be established in social services encounters between staff and users and point out that this is a social process in which the importance of establishing a mutual trust holds the process together. The importance of establishing mutual trust is often expressed in previous studies (Denhov & Topor, 2012; Marttila et al., 2012; Topor et al., 2018). The present results are consistent with previous research emphasizing continuity and a possibility for users to receive a second chance in life (Denhov & Topor, 2012; Marttila et al., 2012; Nehlin et al., 2020; Topor et al., 2018). Earlier studies illustrate how establishing trust is perceived by staff members as a dilemma due to their dual role of being tough decision makers on the one hand and supportive on the other (Marttila et al., 2012). In addition, personnel perceive this as a process that starts disadvantaged, because users are being suspicious of staff and therefore the process takes time. Likewise, the staff expressed suspicion of users (ibid). The present model sheds light on this process through a user perspective which is in alignment with previous study by Marttila et al. (2012).

In previous research most service users with an alcohol use disorder and mental illness prefer having an active role in the decision-making process (Liebherz, Härter et al., 2015; Liebherz, Tlach et al., 2015). This finding is in line with our results; even if temporary pauses of participation are described, and reliable staff substitutes the user’s voice in planning and decision making (Laitila et al., 2011; Thompson, 2007). Regardless of these pauses, being respected and treated as an active partner are deemed essential by users.

The framework for user participation needs further testing with a larger sample. In addition, the framework needs testing in practice as what is said regarding the encounters can deviate from what is going on. Moreover, relevant for the future would be to explore how mutual trust is established in social work encounters according to both users and staff, and to examine further those factors that hinder and facilitate mutual trust, communication and reciprocity.

## Conclusion

The study illustrates how people with comorbidity are carriers of a valuable experience-based knowledge that can be considered indispensable in an evidence-based practice. The ability to participate and impart this knowledge can fluctuate due to periods of mental illness or ongoing addiction. The constructed model shows that mutual respect for each other’s knowledge and willingness for user participation of both users and social workers are important keys to such practice.
